# Application and effect of developmental supportive care on growth and neurobehavioral development in preterm infants

**DOI:** 10.3389/fped.2025.1738380

**Published:** 2025-12-17

**Authors:** Xiaoqing Niu, Ling Li, Yachao Jing

**Affiliations:** 1 Department of Pediatrics, Zhangjiakou First Hospital, Zhangjiakou, Hebei, China; 2 Department of Obstetrics, Zhangjiakou First Hospital, Zhangjiakou, Hebei, China; 3 Department of Gynecology (East Courtyard) Ward, Zhangjiakou First Hospital, Zhangjiakou, Hebei, China

**Keywords:** cognitive development, developmental supportive care, growth and development, neurobehavioral function, preterm infants

## Abstract

**Objective:**

To evaluate the effects of developmental supportive care on growth, neurobehavioral function, and cognitive development in preterm infants.

**Methods:**

In this retrospective study, a total of 104 preterm infants born in our hospital were enrolled and divided into the routine care group (*n* = 49) and the developmental supportive care group (*n* = 55) based on the nursing approach they received during their hospitalization. Both groups received nursing care during hospitalization for 7 days. Routine care included monitoring of vital signs, feeding management, environmental control, skin and oral care, positioning, management of clinical conditions, and parental education. Developmental supportive care was implemented on the basis of routine care and comprised individualized care plans, environmental and sensory regulation, clustered care, tactile stimulation, non-nutritive sucking training, kangaroo care, feeding optimization, pain and stress management, parental involvement, and systematic recording and evaluation of the care effects. Outcome measures included body weight, length, head circumference, and chest circumference before and after the care; neurobehavioral function (passive muscle tone, active muscle tone, primitive reflexes, behavioral state, and general status); and cognitive development assessed by the Mental Development Index (MDI) and Psychomotor Development Index (PDI).

**Results:**

There were no statistically significant differences between the two groups in baseline characteristics, growth indices, neurobehavioral function, or cognitive development before the care (*P* > 0.05). After the care, growth parameters increased in both groups, with body weight, length, head circumference, and chest circumference significantly higher in the developmental supportive care group than in the routine care group (*P* < 0.05). Neurobehavioral scores as well as MDI and PDI scores improved in both groups, with significantly greater improvements observed in the developmental supportive care group (*P* < 0.05).

**Conclusions:**

Developmental supportive care can effectively promote growth, neurobehavioral function, and cognitive development in preterm infants. Compared with routine care, this model offers systematic and individualized nursing approaches that enhance physiological stability and developmental outcomes, providing reliable evidence for clinical neonatal nursing practice.

## Introduction

Due to their insufficient gestational age and immature organ development, preterm infants are prone to physiological instability, low immunity, and abnormalities in neurobehavioral development (1, 2). Their growth and neurobehavioral functions are influenced by multiple factors. Although routine neonatal care provides basic life support and daily nursing, it lacks systematic interventions tailored to the developmental characteristics of preterm infants, making it difficult to fully promote their physical growth and neurobehavioral development (3, 4).

Developmental supportive care (DSC) is a nursing model tailored to the physiological and neurodevelopmental characteristics of preterm infants (5, 6). Through individualized assessment, environmental and sensory regulation, optimization of positioning and bedding, clustered care, tactile stimulation, non-nutritive sucking training, and skin-to-skin contact. DSC aims to reduce stress responses, improve physiological stability, and promote neurobehavioral and cognitive development, while enhancing parental involvement and parent–infant interaction (7). Existing studies have shown that DSC has potential advantages in improving the growth, neurobehavioral, and cognitive-motor development of preterm infants; however, its clinical effectiveness still requires systematic evaluation (8, 9).

This retrospective study aimed to systematically evaluate the efficacy of developmental supportive care in promoting growth, neurobehavioral function, and cognitive development in preterm infants. We hypothesized that DSC would yield superior outcomes compared to routine care alone. The findings are expected to provide clinical evidence for optimizing neonatal nursing practices and improving long-term developmental outcomes in this vulnerable population.

## Materials and methods

### General information

In this retrospective study, a total of 104 preterm infants born in our hospital between January 2023 and December 2023 were enrolled. No *a priori* sample size calculation was performed due to the retrospective nature of the study. All eligible infants admitted during the study period were included to minimize selection bias. Based on the nursing approach they received during their hospitalization, they were divided into the routine care group (*n* = 49) and the developmental supportive care group (*n* = 55). Group assignment was not randomized but was determined by the clinical practice and nursing schedule in effect during the infant's hospitalization period. All infants were hospitalized in the Neonatal Intensive Care Unit (NICU) throughout the study period. The care commenced within 72 h after birth and continued for 7 days during NICU hospitalization. Baseline assessments were conducted before care initiation. Post-care evaluations for growth and neurobehavioral function were performed at the end of the 7 days hospitalization period. Cognitive development was assessed at a follow-up visit when infants reached a corrected age of 40 weeks. This study was approved by the Ethics Committee of Zhangjiakou First Hospital (Approval No. 2025-LW-32). Informed consent was obtained from the guardians of all newborns included in the study.

**Inclusion criteria:** Preterm infants meeting the diagnostic criteria for late preterm birth, with a gestational age of 34 ≤ 37 weeks; admitted within 72 h after birth; complete clinical data available.

**Figure 1:**
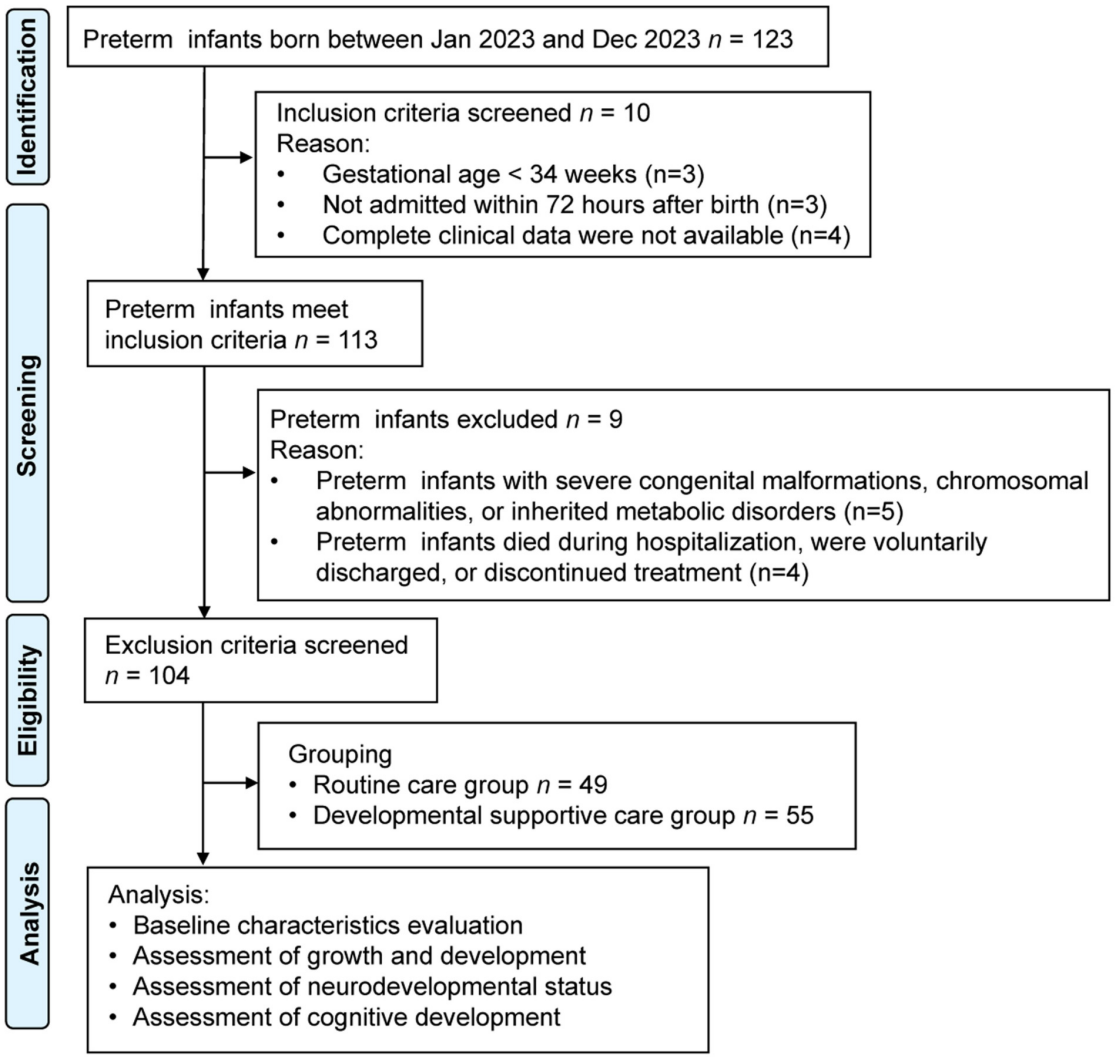
Flow chart of patient screening.

**Exclusion criteria:** Infants with gestational age >34 weeks who have severe congenital malformations, chromosomal abnormalities, or inherited metabolic disorders. Death during hospitalization, voluntary discharge, or discontinuation of treatment (Figure 1).

### Nursing methods

The routine care group received routine neonatal care during hospitalization. This included: (1) Routine monitoring: Daily assessment of vital signs, including body temperature, respiration, heart rate, and oxygen saturation; routine laboratory tests such as blood glucose and blood gas analysis were performed as prescribed. (2) Basic feeding care: Infants were fed breast milk or formula according to medical orders; tube feeding was performed when necessary. Position changes and back patting were conducted on schedule to assist with gas expulsion. (3) Environmental management: The ward environment was maintained at appropriate temperature (24℃–26℃) and humidity (50%–60%), kept quiet and clean, with strict adherence to hand hygiene and aseptic procedures. (4) Routine skin and oral care: Skin cleaning, diaper changes, and umbilical cord care were performed daily or per shift according to the nursing pathway. Oral care was provided as needed, using gauze or cotton swabs for gentle wiping. (5) Routine positioning and bedding: Positions (supine or lateral) were changed as clinically required, without specialized “nest-style” supports or positioning aids. (6) Disease management: Abnormal conditions such as apnea, pallor, or vomiting were promptly reported and managed. (7) Health education and psychological care: Parents received education on routine neonatal care to reduce anxiety.

The developmental supportive care group received developmental supportive care in addition to routine neonatal care during hospitalization. The developmental supportive care was implemented by a multidisciplinary team including NICU nurses, neonatologists, physical therapists, and nutritionists. Regular team meetings were conducted to ensure consistent application of DSC principles and coordinate care activities. The NICU nurses responsible for delivering DSC had received specialized, advanced training in developmental care principles. This training comprised a structured 20-h program covering the theoretical foundations of developmental supportive care, practical care techniques (such as positioning, tactile stimulation, and pain management), and competency-based assessment. Furthermore, all these nurses held a bachelor's degree or higher in nursing and had a minimum of two years of NICU experience.

The care period lasted 7 days. Specific measures are summarized in Table 1.

**Table 1:** Components of developmental supportive care measure.

Component	Description	Frequency/Duration
Individualized Assessment	Comprehensive evaluation of gestational age, physiological stability, and neurobehavioral status	Daily
Environmental Regulation	Light and noise control, maintained temperature (24°C–26°C) and humidity (50°C–60%)	Continuous
Positioning	Nest-style bedding with boundary support	Adjusted as needed
Clustered Care	Procedure grouping to minimize disturbances	During care cycles
Tactile Stimulation	Gentle touch and massage	3–5 min, 1–3 times daily
Non-nutritive Sucking	Oral motor training	3–5 min, 1–3 times daily
Kangaroo Care	Skin-to-skin contact	30–60 min as tolerated
Feeding Optimization	Breastfeeding support and oral stimulation	During feeding times
Pain Management	Non-pharmacological soothing techniques	As needed
Parental Education	Hands-on training in DSC techniques	Daily sessions

Parental involvement was actively encouraged and structured. Parents received comprehensive education on DSC principles and techniques, including hands-on training in kangaroo care, feeding optimization, and behavioral observation. They were guided to participate in daily care activities and record infant responses under nursing supervision.

### Observation indicators

The observation indicators included:

Growth parameters: Changes in body weight, length, head circumference, and chest circumference after the care were recorded and compared with pre-care values and the routine care group to evaluate the effect of the care on the growth and development of preterm infants.
Neurobehavioral function: Neurobehavioral function was assessed using the Neonatal Behavioral Assessment Scale (NBAS), which evaluates passive muscle tone, active muscle tone, primitive reflexes, behavioral state, and general condition. Assessments were conducted by two trained neonatologists (10). Inter-rater reliability was maintained at >90% through regular calibration sessions. The NBAS assessment was performed at baseline and upon completion of the 7 days care period.
Cognitive and motor development: Cognitive and motor development were assessed using the Bayley Scales of Infant and Toddler Development, Third Edition (Bayley-III), which is validated for use in preterm infants from 1 to 42 months of age (11). The Mental Development Index (MDI) and Psychomotor Development Index (PDI) of preterm infants were used to evaluate. The MDI mainly assesses cognitive abilities (attention, visual tracking, hand–eye coordination, exploratory behavior), language abilities (auditory comprehension and expressive language), memory and learning (object permanence, understanding cause–effect relationships), and social behavior (responses to facial expressions and voices, emotional interactions). The PDI primarily evaluates gross motor skills (head and neck control, rolling over, sitting, crawling, standing, walking) and fine motor skills (grasping, finger manipulation, hand–eye coordination). Each item is scored based on the infant's performance, and cumulative scores are converted to standard scores (mean 100, SD 15). Scores reflect developmental levels: 85–115, normal; 70–84, mild delay; 55–69, moderate delay; <55, severe delay. Changes in MDI and PDI scores before the care and at 40 weeks of corrected gestational age were compared between the two groups to observe the effect of developmental supportive care on cognitive development.

### Statistical analysis

Statistical analyses were performed using SPSS version 27.0. Continuous variables were tested for normality using Shapiro–Wilk test and are presented as mean ± standard deviation. Categorical data are presented as frequencies and percentages (*n*/%). Between-group comparisons of baseline characteristics and outcome measures were analyzed using independent *t*-tests for continuous variables and *χ*² tests for categorical variables. Within-group changes from baseline to post-intervention were analyzed using paired *t*-tests. A *p*-value of <0.05 was considered statistically significant.

## Results

### Comparison of baseline characteristics

The baseline demographic and clinical characteristics of the preterm infants in both groups are presented in Table 2. There were no statistically significant differences between the routine and developmental supportive care groups in terms of gender, mode of delivery, Apgar score, or incidence of complications (all *P* > 0.05), indicating that the two groups were comparable before the care. Most importantly, there was no significant difference in gestational age between the two groups (*P* = 0.941). This baseline comparability in the most critical determinant of newborn growth and development strengthens the validity of directly comparing post-care outcomes between the groups (Table 2).

**Table 2:** Comparison of baseline characteristics between the two groups.

Factors	Routine care group (*n* = 49)	Developmental supportive care group (*n* = 55)	*χ*^2^/t	*P*
Gender (*n*/%)				
Male	23 (46.94)	27 (49.09)	0.048	0.826
Female	26 (53.06)	28 (50.91)		
Gestational age (weeks)	33.52 ± 3.82	33.47 ± 3.78	0.074	0.941
Mode of delivery (*n*/%)				
Vaginal delivery	13 (26.53)	16 (29.09)	0.084	0.771
Cesarean section	36 (73.47)	39 (70.91)		
Apgar score	4.74 ± 1.23	4.67 ± 1.03	0.341	0.734
Complications				
Neonatal asphyxia (*n*/%)				
Yes	5 (10.20)	8 (14.55)	0.447	0.504
No	44 (89.80)	47 (85.45)		
Neonatal pneumonia (*n*/%)				
Yes	13 (26.53)	16 (29.09)	0.084	0.771
No	36 (73.47)	39 (70.91)		
Intracranial hemorrhage (*n*/%)				
Yes	10 (20.41)	12 (21.82)	0.031	0.860
No	39 (79.59)	43 (78.18)		

### Comparison of growth and development between the two groups of neonates

Developmental supportive care significantly enhanced growth parameters in preterm infants compared to routine care. After the 7 days care period, all growth parameters increased in both groups (all *P* < 0.05). Moreover, the developmental supportive care group demonstrated substantially greater improvements in body weight (2.28 ± 0.25 kg vs. 2.12 ± 0.40 kg, *P* = 0.011), length (46.76 ± 2.76 cm vs. 44.59 ± 2.66 cm, *P* < 0.001), head circumference (32.61 ± 1.68 cm vs. 31.52 ± 1.65 cm, *P* = 0.001), and chest circumference (33.71 ± 2.91 cm vs. 31.58 ± 3.14 cm, *P* < 0.001) (Table 3).

**Table 3:** Comparison of growth and development between the two groups of neonates.

Factor	Routine care group (*n* = 49)	Developmental supportive care group (*n* = 55)	χ^2^/*t*	*P*
Pre-care body weight (kg)	1.77 ± 0.36	1.84 ± 0.34	1.137	0.258
Post-care body weight (kg)	2.12 ± 0.40	2.28 ± 0.25	2.588	0.011
Pre-care body length (cm)	42.20 ± 2.83	43.10 ± 2.68	1.655	0.101
Post-care body length (cm)	44.59 ± 2.66	46.76 ± 2.76	4.059	<0.001
Pre-care head circumference (cm)	29.57 ± 1.73	29.86 ± 1.69	0.845	0.400
Post-care head circumference (cm)	31.52 ± 1.65	32.61 ± 1.68	3.318	0.001
Pre-care chest circumference (cm)	30.70 ± 2.74	31.10 ± 2.93	0.722	0.472
Post-care chest circumference (cm)	31.58 ± 3.14	33.71 ± 2.91	3.590	<0.001

### Comparison of neurodevelopment between the two groups of neonates

Developmental supportive care group showed significantly greater improvements in neurobehavioral outcomes across all measured domains. After the care, all these neurodevelopmental indicators improved in both groups (all *P* < 0.05). Moreover, developmental supportive care resulted in superior scores in passive muscle tone (5.80 ± 1.20 vs. 4.50 ± 0.86, *P* < 0.001), behavioral state (8.58 ± 1.21 vs. 6.99 ± 1.26, *P* < 0.001), primitive reflexes (5.26 ± 0.68 vs. 4.26 ± 0.99, *P* < 0.001), active muscle tone (6.21 ± 1.03 vs. 5.03 ± 1.00, *P* < 0.001), and general condition (5.04 ± 0.90 vs. 4.20 ± 0.83, *P* < 0.001) compared to the routine care group (Table 4).

**Table 4:** Comparison of neurodevelopmental Status between the two groups of neonates.

Factors	Routine care group (*n* = 49)	Developmental supportive care group (*n* = 55)	χ^2^/t	*P*
Pre-care passive muscle tone	2.53 ± 1.05	2.55 ± 1.14	0.110	0.913
Post-care passive muscle tone	4.50 ± 0.86	5.80 ± 1.20	6.277	<0.001
Pre-care behavioral ability	4.64 ± 1.46	4.75 ± 1.47	0.403	0.688
Post-care behavioral ability	6.99 ± 1.26	8.58 ± 1.21	6.581	<0.001
Pre-care primitive reflexes	2.04 ± 0.93	2.09 ± 1.01	0.241	0.810
Post-care primitive reflexes	4.26 ± 0.99	5.26 ± 0.68	6.045	<0.001
Pre-care active muscle tone	3.22 ± 0.40	3.20 ± 0.42	0.242	0.809
Post-care active muscle tone	5.03 ± 1.00	6.21 ± 1.03	5.950	<0.001
Pre-care general condition	2.68 ± 0.71	2.70 ± 0.75	0.198	0.843
Post-care general condition	4.20 ± 0.83	5.04 ± 0.90	4.909	<0.001

### Comparison of cognitive development between the two groups

Developmental supportive care significantly enhanced cognitive and psychomotor development in preterm infants. After the care, both MDI and PDI scores increased in both groups (all *P* < 0.05). Moreover, the developmental supportive care group achieved markedly higher MDI scores (90.89 ± 5.33 vs. 81.30 ± 4.89, *P* < 0.001) and PDI scores (91.98 ± 6.18 vs. 87.13 ± 5.92, *P* < 0.001) following the intervention period compared to the routine care group (*P* < 0.05) (Table 5).

**Table 5:** Comparison of cognitive development between the two groups.

Factors	Routine care group (*n* = 49)	Developmental supportive care group (*n* = 55)	χ^2^/t	*P*
Pre-care MDI score	72.43 ± 5.33	73.36 ± 5.06	0.917	0.361
Post-care MDI score	81.30 ± 4.89	90.89 ± 5.33	9.528	<0.001
Pre-care PDI score	80.99 ± 6.11	81.32 ± 5.98	0.285	0.776
Post-care PDI score	87.13 ± 5.92	91.98 ± 6.18	4.076	<0.001

## Discussion

This study demonstrates that developmental supportive care significantly promotes growth, neurobehavioral function, and cognitive development in preterm infants. After receiving developmental supportive care during hospitalization, infants in the developmental supportive care group showed significantly greater increases in body weight, length, head circumference, and chest circumference compared with the routine care group, indicating that this care model effectively improves physical growth in preterm infants. These findings are consistent with previous studies. Fawzia et al. reported that developmental supportive care, by reducing unnecessary stimuli and optimizing positioning and environmental conditions, can significantly enhance weight gain and physiological stability in preterm infants (12). Similarly, some researchers found that individualized nursing measures improve energy utilization efficiency in preterm infants and promote physical development, which aligns closely with the results of the present study (13, 14).

The superior outcomes observed in the developmental supportive care group can be attributed to several mechanistic factors. DSC reduces physiological stress by minimizing environmental stimuli and providing comforting boundaries, thereby conserving energy for growth and development (15). The enhanced tactile stimulation and kangaroo care promote vagal activity and release of growth factors, supporting both physical growth and neural maturation (16). Furthermore, clustered care practices minimize sleep disruption, allowing for more organized sleep-wake cycles that are crucial for brain development (17, 18). The parental involvement component not only provides emotional security but also enhances the infant's social engagement, forming the foundation for cognitive development (19).

In terms of neurobehavioral development, this study found that the developmental supportive care group exhibited superior passive muscle tone, active muscle tone, primitive reflexes, behavioral state, and general condition compared with the routine care group. Previous studies have observed significant improvements in behavioral state and neurobehavioral scores in preterm infants receiving kangaroo mother care in the NICU, highlighting the importance of tactile stimulation, non-nutritive sucking training, and skin-to-skin contact for neurological maturation (20, 21). Pavlyshyn et al. further emphasized that clustered care and reduced frequency of awakenings can lower stress levels, thereby promoting early neurobehavioral development in preterm infants (22). The findings of the present study align with these studies, indicating that systematic and individualized developmental supportive care can improve neurobehavioral function in preterm infants in the short term (23, 24).

In terms of cognitive development, the developmental supportive care group showed significantly higher MDI and PDI scores compared with the routine care group, indicating that developmental supportive care has a positive effect on the cognitive and motor development of preterm infants (25). Consistent with these findings, Carnevali et al. reported that early interventions for preterm infants, including regular tactile stimulation and skin-to-skin contact, can improve MDI and PDI scores, with particularly notable effects on gross and fine motor development (26). Furthermore, study has shown that parental involvement and parent–infant interaction positively influence cognitive and language development (27, 28). In this study, improvements in cognitive development were also observed through kangaroo care and parental participation, further supporting the effectiveness of developmental supportive care (29, 30).

Although the results of this study demonstrate that developmental supportive care offers significant advantages in the care of preterm infants, several limitations remain. First, the sample size was limited and the study was conducted at a single center, which may affect the generalizability of the findings. Second, its retrospective and non-randomized design introduces the potential for selection bias and unmeasured confounding factors. Although baseline characteristics were comparable between the two groups, we cannot rule out the possibility that other unmeasured variables influenced the outcomes. Third, the care period was relatively short (only 7–10 days during hospitalization), preventing assessment of long-term growth, neurobehavioral, and developmental outcomes. Future studies with extended intervention durations and longer follow-up periods are needed to evaluate sustained benefits. Although the baseline characteristics of the two groups were comparable, particularly gestational age, this greatly reduced the possibility of confounding results due to differences in maturity. However, the lack of statistical adjustment for age remains an inherent limitation of the retrospective design, and future prospective studies with prespecified adjustment models are needed to confirm our conclusions. Additionally, this study did not analyze the independent effects of different components within the care protocol, making it difficult to determine the specific contribution of each measure. Future research could adopt multicenter, large-sample designs, extend follow-up periods, and use grouping or factor analysis to further investigate the independent effects of each intervention on the growth, neurobehavioral function, and cognitive development of preterm infants.

In summary, the findings of this study indicate that developmental supportive care, through individualized and systematic cares, significantly enhances growth, neurobehavioral function, and cognitive development in preterm infants. These results are consistent with previous domestic and international studies, providing reliable evidence for evidence-based clinical care of preterm infants.

## Data Availability

The raw data supporting the conclusions of this article will be made available by the authors, without undue reservation.
